# First Report of Diabetes Phenotype due to a Loss-of-Function* ABCC8* Mutation Previously Known to Cause Congenital Hyperinsulinism

**DOI:** 10.1155/2019/3654618

**Published:** 2019-04-11

**Authors:** Theocharis Koufakis, Amalia Sertedaki, Elizabeth-Barbara Tatsi, Christina-Maria Trakatelli, Spyridon N. Karras, Eleni Manthou, Christina Kanaka-Gantenbein, Kalliopi Kotsa

**Affiliations:** ^1^Division of Endocrinology and Metabolism and Diabetes Center, First Department of Internal Medicine, Medical School, Aristotle University of Thessaloniki, AHEPA University Hospital, Thessaloniki, Greece; ^2^Division of Endocrinology, Metabolism and Diabetes, First Department of Pediatrics, Medical School, National and Kapodistrian University of Athens, “Aghia Sophia” Children's Hospital, Athens, Greece

## Abstract

Monogenic Diabetes is relatively rare, representing only 1-2% of total diabetes cases; nevertheless, it is often misdiagnosed primarily as type 1 diabetes, leading to unnecessary insulin therapy and delayed recognition of affected family members. In the present article, we describe a case of a young, male patient who presented with hyperglycemia in the absence of ketosis and following genetic testing; he proved to harbor the loss-of-function p.Arg1353His (c.4058G>A) mutation in the* ABCC8* gene, inherited from his mother. This mutation has been previously described in patients with Congenital Hyperinsulinism. Furthermore, different mutations in the* ABCC8* gene have been linked with MODY 12, type 2, and gestational diabetes; however, to the best of our knowledge, this is the first report that associates this specific mutation with diabetes phenotype.* ABCC8*-related diabetes is characterized by remarkable heterogeneity in terms of clinical presentation and therapeutic approach. Early diagnosis and individualized treatment are essential to achieving metabolic targets and avoiding long-term diabetes complications.

## 1. Introduction

Diabetes Mellitus (DM) is a heterogenous group of disorders, mainly characterized by high blood glucose concentrations and metabolic derangement, as a result of beta-cell insufficiency, deficits in insulin secretion and action, or a combination of the above [1]. The risk for developing DM is—to a certain extent—genetically determined; still, different diabetes types follow different inheritance patterns. Type 1 diabetes (T1D), type 2 diabetes (T2D), Latent Autoimmune Diabetes in Adults (LADA), and Gestational Diabetes Mellitus (GDM) are considered as polygenic, multifactorial diseases, since their development is a result of complex interactions between genetic, environmental, and lifestyle components [2]. On the contrary, Neonatal Diabetes Mellitus (NDM), Syndromic Diabetes, and Maturity-Onset Diabetes of the Young (MODY) are monogenic entities, caused by highly penetrant inherited or sporadic mutations in genes playing an important role in beta‐cell function [3, 4]. These forms, following the new terminology, are now under the definition of Monogenic Diabetes [5]. Monogenic Diabetes is relatively rare, representing only 1-2% of total diabetes cases; however, it is often misdiagnosed primarily as T1D, leading to unnecessary insulin therapy and delayed recognition of affected family members [6].

The ATP-binding cassette transporter subfamily C member 8 (*ABCC8*) gene is located on chromosome 11p15.1, containing 39 exons and encoding the sulfonylurea receptor 1 (SUR1) protein [7]. SUR1 belongs to ATP-binding cassette transporters that promote ATP hydrolysis to transport substrates. However, SUR1 is not directly involved to substrates transportation but mainly adjusts the activity of Kir6.2 in the K_ATP_ channel compound [8].

Mutations in potassium voltage-gated channel subfamily J member 11 (*KCNJ11*) and* ABCC8* genes are known to cause two contrasting phenotypes, which are Congenital Hyperinsulinism (CH) and NDM. Loss-of-function (inactivating) mutations abrogate the channel function causing CH, whereas gain-of-function (activating) mutations disrupt the ability of adenosine triphosphate (ATP) to close the channel, resulting in NDM [9]. Patients with monoallelic mutations of* ABCC8* typically present with complete loss of K_ATP_ channel function, thus resulting in severe forms of diazoxide-unresponsive CH [10].* ABCC8* gene mutations also constitute common genetic etiology of Permanent and Transient DM [11]. Recently ABCC8 has been recognized as the MODY12 subtype with an increasing number of relative reports [12–16].

In the present article, we describe a case of a young patient who presented with hyperglycemia in the absence of ketosis and following genetic testing, he proved to be a carrier of the p.Arg1353His mutation in the* ABCC8* gene, inherited from his mother. To the best of our knowledge, this is the first report that associates this specific mutation with such a phenotype. 

## 2. Clinical Report

**Figure 1:**
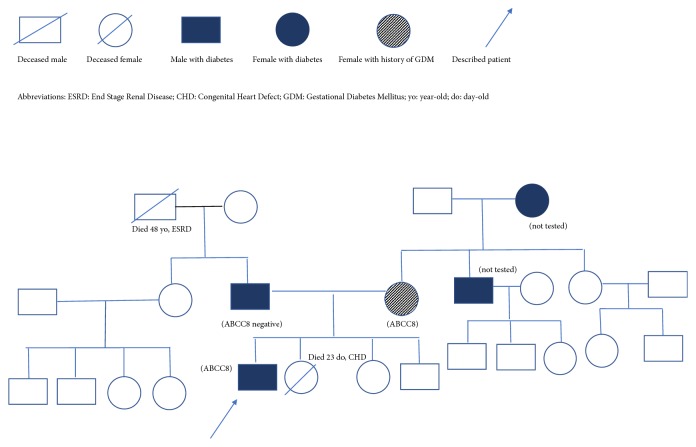
Pedigree chart of the described family.

A 17-year-old Caucasian male, presented to the Emergency Department, with complaints of polydipsia, polyuria, and xerostomia, for the last 30 days. He also reported a weight loss of about 7 kg during the same time period. He had an unremarkable birth and development history. He was born via spontaneous delivery at 39 weeks of gestation, with a body weight of 3400 g (54^th^ percentile), length of 50 cm (52^nd^ percentile), and head circumference of 34 cm (36^th^ percentile). He was the first child of the family, while his siblings, aged 13 (female) and 3 (male) years, respectively, were healthy. The second (female) child of the family succumbed 23 days after birth, due to congenital heart defect (Tetralogy of Fallot). Patient's mother, aged 45, reported a history of diet-controlled GDM during her last pregnancy and his father, aged 50, had a history of diet-controlled T2D, diagnosed at the age of 45. There was no history of neonatal hyperinsulinism in the family. The rest of his family history included adult-onset DM diagnosed in patient's grandmother and uncle, both from maternal side (Figure 1).

At presentation, patient's physical examination was normal. He weighed 68 kg (Body Mass Index 22.7 kg/m^2^), his office Blood Pressure was 109/60 mm Hg, and his pulse rate was 84/min. Laboratory evaluation revealed high, fasting plasma glucose (FPG) concentrations (246 mg/dl/13.65 mmol/L), glycated hemoglobin A1c levels (HbA1_C_) of 9.9% (84.7 mmol/mol), absence of ketones in urine, and normal arterial pH (7.40). C-peptide and fasting insulin levels were ordered and proved to be within the low normal range [1.84 ng/ml (reference range 1.1-4.4) and 8.3 *μ*IU/ml (reference range 2.6-24.9), respectively]. Testing for islet cell cytoplasmic autoantibodies (ICA), Glutamic Acid Decarboxylase Autoantibodies (GADA), and Insulin Autoantibodies (IAA) produced negative results. The combination of negative autoantibodies for T1D with inappropriately low C-peptide and insulin levels and in the absence of ketosis, along with a family history of diabetes, prompted the molecular investigation for MODY.

## 3. Genetic Testing

DNA was isolated from peripheral blood leukocytes employing the Maxwell® 16 Blood DNA Purification Kit (Promega, Madison, WI, USA) according to the manufacturer's instructions.

Patient's DNA was tested for mutations in seven MODY genes,* GCK, HNF1A, HNF4A*,* HNF1B, INS, ABCC8, *and* KCNJ11*, employing a Next Generation Sequencing (NGS) targeted gene panel on an Ion Torrent™ Personal Genome Machine™ (PGM) platform (Thermo Fisher Scientific, Waltham, MA, USA) using the Ion PGM™ Hi-Q™ View Sequencing Kit and ion 314™ chip v2. The panel had a size of 29.45kb containing 110 amplicons and covering 98.87% of the targeted regions. The primers used were designed by the Thermo Fisher Scientific Ion AmpliSeq Designer platform (version 5.6; www.ampliseq.com). Following sequencing, base calling, alignment of the amplicons, and variant calling were performed on the Torrent Suite™ Server using the instrument's default settings. The sequencing data were aligned to the human genome reference hg19 with Torrent Mapping Alignment Program (TMAP). The variants were annotated by the Ion Reporter software (v5.2.0.66; https://ionreporter.thermofisher.com) and by the ANNOVAR (http://annovar.openbioinformatics.org) through VarAFT software (Variant Annotation and Filter Tool, Version 2.05; https://varaft.eu/).

Sanger sequencing of exon 33 of the* ABCC8* gene (GenBank_NM00352.2) was carried out employing the BigDye™ Terminator v3.1 Cycle Sequencing Kit on an Applied Biosystems 3500 Genetic Analyzer (Applied Biosystems, USA).

Written informed consent was obtained by the patient and his parents. The study was approved by the Institutional Scientific Committee and is in accordance with the Declaration of Helsinki.

## 4. Results of Genetic Testing

Due to the nonspecific MODY phenotype of our patient a NGS targeted gene panel was chosen to search for mutations in seven MODY genes,* GCK, HNF1A, HNF4A*,* HNF1B, INS, ABCC8, *and* KCNJ11*. The patient was found to harbor the previously described p.Arg1353His* ABCC8* gene mutation. This is a G to A transition at position c.4058, substituting Arginine to Histidine at codon 1353 in exon 33 of the* ABCC8* gene. Sanger sequencing verified the presence of the mutation in the patient and his mother, but not in his father. The Arginine 1353 residue was found to be conserved in various species, as in Pan Troglodyte, Macaca mulatta, Felis Catus, Mus Musculus, Gallus Gallus, Takifugu Rubripes, Danio Regio, and Caenorhabditis Elegans.

## 5. Follow-Up

The patient was put on combination therapy with basal (glargine) insulin and a sulfonylurea (SU) (glibenclamide 5 mg three times daily). In a follow-up visit after three months on this regimen, his glycemic control had remarkably improved, with the vast majority of both fasting and postprandial glucose values within the target range and an HbA1_C_ level of 6.7% (49.7 mmol/mol). At that time, an Oral Glucose Tolerance Test was ordered for his mother with normal results. We have already planned to proceed in genetic testing of the other members of the family with diabetes, at the earliest opportunity.

## 6. Discussion

The presented case demonstrates the clinical presentation and diagnosis of diabetes in an adolescent patient and his family, associated with the p.Arg1353His mutation in the* ABCC8* gene. The same mutation resulted in the development of gestational diabetes in the mother and of an unclassified diabetes phenotype in the male offspring. NGS enabled rapid and cost-effective diagnosis, compared to sequential Sanger sequencing of various putative phenotype related genes and taking into consideration the large size of the* ABCC8* gene [19].

This mutation has been previously described in a case of familial Leucine-Sensitive Hypoglycemia [20], a form of CH. Functional analysis and electrophysiological studies of the mutant channels revealed that the p.Arg1353His mutation causes partial disruption of SUR1 receptor function [20]. Khoriati et al. [21] described the existence of the same* ABCC8* mutation in a male infant with prematurity, hyperinsulinemic hypoglycemia, and macrosomia. None of these features were present in our patient. Calabria et al. [22] studied nine subjects with CH due to inactivating mutations in the K_ATP_ channel (among them, a 47 year-old-female was a carrier of the p.Arg1353His mutation). Three of these patients had been subjected to pancreatectomy in order hypoglycemia to be controlled, whereas none of the subjects were on medical therapy for hyperinsulinism at the time of the study. It was demonstrated that the Glucagon-like Peptide-1 (GLP-1) receptor agonist exendin-(9-39) significantly increased mean nadir blood glucose and glucose area under the curve, suggesting the GLP-1 receptor as a potential therapeutic target for K_ATP_-related CH.

*ABCC8* mutations have been associated with Monogenic Diabetes (MODY12), T2D, type 1b (idiopathic) diabetes, and GDM (Table 1). The phenotype of MODY12 patients exhibits great variability. Most patients present clinical features similar to MODY1 and MODY3 [14]. Surprisingly, an* ABCC8* mutation has been reported in a 3-generation pedigree with a MODY2 resembling phenotype of mild hyperglycemia and no need of pharmacological treatment [13]. A family with a very heterogeneous nature of diabetes and a gain-of-function mutation in the* ABCC8* gene has been recently reported [18]. The diabetes phenotype of the mutation carriers ranged from transient NDM, to insulin dependent diabetes and Τ2D, suggesting a genetic anticipation-like phenotype in genetic diabetes. This phenotype variability can be the consequence of the type and the location of the mutation itself, along with the interplay between other modifying genetic and environmental factors.

Riveline et al. aimed to determine the clinical and metabolic features of adult-onset diabetes caused by gain-of-function* ABCC8* mutations, among members of NDM families, a Monogenic Diabetes form too [17]. They found that these features were diverse, ranging from principally glucose intolerance to frank diabetes or insulin-requiring diabetes since diagnosis. Impaired insulin secretion capacity was shown in three mutation carriers compared to adult controls, while in two of them, this was restored after treatment with SU. As shown by Pearson et al. in patients with heterozygous activating Kir6.2 mutations in* KCNJ11* [23], in the absence of SUs, the beta-cell membrane is hyperpolarized, resulting in blockage of beta-cell's response to incretins and other stimuli. SUs close the K_ATP_ channel and depolarize the membrane and through this mechanism, they restore beta cells' ability to respond to GLP-1 or other secretagogues [17].

The most interesting finding of the present report is that a loss-of-function mutation in the* ABCC8* gene previously known to cause CH was detected in a patient and his mother, presenting features of adult-onset and gestational diabetes, respectively. It is well known that some individuals suffering from CH in early life later develop diabetes (even in the absence of pancreatectomy). However, this was not the case in the described family, given that both individuals had unremarkable birth and development histories. The mechanism through which K_ATP_ defects cause diabetes in patients with loss-of-function mutations is believed to be beta-cell apoptosis as a consequence of continuous depolarization and elevated cytoplasmic calcium concentrations within the beta-cell [24]. Further studies are needed to elucidate the complex underlying gene-gene and gene-environment interactions resulting in the remarkable phenotypic heterogeneity related to* ABCC8* p.Arg1353His mutation.

In conclusion, the described case highlights the heterogenous clinical expression of diabetes related to* ABCC8* gene and underlines the value of genetic testing in young patients presenting with nonautoimmune diabetes. This is facilitated by the utility of the NGS methodology targeted gene panel, which is becoming the method of choice for clinical diagnosis for patients with a nonspecific MODY phenotype. Early diagnosis and individualized treatment are essential to achieving metabolic targets and avoiding long-term diabetes complications.

## Figures and Tables

**Table 1:** Main findings of key studies that reported the presence of mutations in the *ABCC8 *gene in people with diabetes. Remarkable heterogeneity in terms of both clinical presentation and treatment is evident.

First author, year [Ref]	Mutation (as reported in the study)	Diabetes Phenotype	Treatment
Gonsorcikova, 2011 [13]	V84I	19 yo m, mild fasting hyperglycemia diagnosed at the age of 12, other family members with the same mutation presented GDM, IFG, IGT	n.r.

Bowman, 2012 [14]	R1380H/N, G214R/V222M, N1245D/N, V1523L/N, Q485R/N, E100K/N	19 yo m, 11 yo f, 15 yo f, 14 yo m, 36 yo m, 13 yo f, 42 yo m (phenotype not specified)	Insulin, SU, metformin or a combination of the above

Riveline, 2012 [17]	R1380H, C435R, L582V, Y356C, P201S, C418R, R620C, R826W	17 yo f diabetes with polyuria, 15 yo m diabetes with polyuria, 36 yo m diabetes, 32 yo m diabetes with obesity, 39 yo m diabetes, 35 yo f IGT, 53 yo f T2D, 53 yo m T2D, 46 yo m T2D, 49 yo f T2D	SU or no treatment

Ovsyannikova, 2016 [12]	Ala1457Thr	28 yo m, hyperglycemia without ketosis, DR and microalbuminuria at diagnosis, mother with the same mutation presented adult onset diabetes	Gliclazide MR plus dapagliflozin

Johnson, 2018 [15]	Ala1390Val	27 yo m, asymptomatic hyperglycemia history of transient NHH, other family members with the same mutation presented adult onset diabetes	Insulin plus gliclazide

Shima, 2018 [18]	V607M/N	Transient neonatal diabetes at birth, other family members with the same mutation presented T2D and T1bD	No treatment

Dallali, 2019 [16]	c.2376delC, c.4606G > A	32 yo m polyuria and polydipsia, 10 yo m fortuitous diagnosis	Insulin, OHA

Koufakis, 2019 [Present article]	p.Arg1353His	17 yo m, hyperglycemia without ketosis, no complications at diagnosis, mother with GDM	Insulin plus glibenclamide

Ref: reference; ABCC8: ATP-binding cassette transporter subfamily C member 8; yo: year-old; m: male; f: female; DR: diabetic retinopathy; MR: modified release; n.r.: not reported; GDM: gestational diabetes mellitus, IFG: impaired fasting glucose; IGT: impaired glucose tolerance; NHH: neonatal hyperinsulinemic hypoglycemia; SU: sulfonylurea; OHA: oral hypoglycemic agents; T2D: type 2 diabetes; T1bD: type 1b diabetes.
